# Exploring the RNA-Recognition Mechanism Using Supervised Molecular Dynamics (SuMD) Simulations: Toward a Rational Design for Ribonucleic-Targeting Molecules?

**DOI:** 10.3389/fchem.2020.00107

**Published:** 2020-02-27

**Authors:** Maicol Bissaro, Mattia Sturlese, Stefano Moro

**Affiliations:** Molecular Modeling Section, Department of Pharmaceutical and Pharmacological Sciences, University of Padova, Padua, Italy

**Keywords:** nucleic acids, RNA, SMIRNA, molecular recognition, molecular dynamics (MD), supervised molecular dynamics (SuMD), structure-based drug design (SBDD)

## Abstract

Although proteins have represented the molecular target of choice in the development of new drug candidates, the pharmaceutical importance of ribonucleic acids has gradually been growing. The increasing availability of structural information has brought to light the existence of peculiar three-dimensional RNA arrangements, which can, contrary to initial expectations, be recognized and selectively modulated through small chemical entities or peptides. The application of classical computational methodologies, such as molecular docking, for the rational development of RNA-binding candidates is, however, complicated by the peculiarities characterizing these macromolecules, such as the marked conformational flexibility, the singular charges distribution, and the relevant role of solvent molecules. In this work, we have thus validated and extended the applicability domain of SuMD, an all-atoms molecular dynamics protocol that allows to accelerate the sampling of molecular recognition events on a nanosecond timescale, to ribonucleotide targets of pharmaceutical interest. In particular, we have proven the methodological ability by reproducing the binding mode of viral or prokaryotic ribonucleic complexes, as well as that of artificially engineered aptamers, with an impressive degree of accuracy.

## Introduction

Ribonucleic acid (RNA) is a polymer whose biological importance has increased progressively over the last 50 years. Despite the central dogma of molecular biology considering this nucleic acid simply as a functional messenger between DNA genetic information storage and protein biosynthesis, RNA has recently been reappraised as an ancestral molecule of primary importance in the abiogenesis process. At the origin of life, RNA probably encompassed both an informational role, which progressively evolved toward involving the more stable and easily replicable DNA polymer, and a catalytic function, which was gradually flanked by more versatile proteins (Morris and Mattick, 2014). The complexity hiding behind RNA's biological functions is intuitable by taking into consideration the human organism, which genetic heritage could quite entirely be transcribed into RNA, despite coding only in a minimal portion (about 3%) for proteins (Warner et al., 2018). A great majority of these transcripts therefore remain untranslated, originating non-coding genomic portions. RNA revolution has thus shed light on the regulatory activity of this widely different class of macromolecules that, along with some proteins, cooperate to control and finely orchestrate the genome expression (Connelly et al., 2016).

RNA polymer lengths range from small hairpins composed of a few tens of nucleobases to long non-coding RNAs sequences (lncRNAs) that can reach up to a few thousands nucleotides (Connelly et al., 2016). Differently from DNA, RNA usually exists as a single-stranded molecule that is not strictly limited by a Watson-Crick base pairing. In solution, ribonucleic acids explore a wide landscape of three-dimensional structures, characterizable by the presence of peculiar functional domains able to specifically recognize other nucleic acids, polypeptides, glyco-derivates, or cognates of small organic molecules (Draper, 1995; Cruz and Westhof, 2009; Salmon et al., 2014; Flynn et al., 2019).

From a topological point of view, the tertiary and quaternary structures that distinguish ribonucleic acids from their deoxyribonucleic counterpart make them more similar to proteins, a consideration that has paved the way for an attempt to pharmacologically modulate their biological functions through the discovery of small molecules interacting with RNA (SMIRNA) (Sucheck and Wong, 2000; Connelly et al., 2016). Interestingly, in recent research work, it has been estimated that pharmacologically modulating RNA would allow us to expanding—by more than an order of magnitude—the universe of targetable macromolecules, and this would thus considerably extend the portion of the druggable genome (Ecker and Griffey, 1999; Warner et al., 2018). Although RNA has been historically considered as an “undruggable” pharmaceutical target, the discovery that many drugs of undeniable therapeutic importance, especially antibiotics, act at this level has attracted the interest of the scientific community, resulting in greater effort being made toward the development of new tools for this purpose (Donlic and Hargrove, 2018; Disney, 2019). Furthermore, the orthogonality characterizing RNA homologous transcripts belonging to virus, prokaryote, and eukaryote genomes make RNA an interesting target for the purpose of achieving selectivity, especially in the field of anti-infectives compound development (Ecker and Griffey, 1999; Connelly et al., 2016). All these aspects, therefore, make the discovery of SMIRNAs extremely intriguing. A first pioneering approach to rationally design new RNA-targeting compounds, simply starting from the knowledge of the oligonucleotide sequence of pathological interest, was developed by the Disney research group and was successfully applied to a plethora of expanded repeating RNAs that are known to cause microsatellite disorders (Velagapudi et al., 2014; Disney et al., 2016). In addition, the quantitative structure-activity relationship (QSAR) model and chemical similarity search were initially exploited to *in-silico* identify or optimize new chemical probes targeting RNA (Disney et al., 2014). Since X-ray crystallography, NMR spectroscopy and, recently, Cryo-EM techniques have unveiled with an atomistic level of detail a multitude of three-dimensional RNA structures, the scientific community has begun to evaluate the applicability of structure-based drug design strategies (SBDD). These approaches, until now mainly validated on proteins targets, could enhance the rational design of SMIRNAs. Molecular docking represents one of the electives of *in silico* techniques, exploited both in the academic and industrial world, to accelerate the discovery and optimization of new drug candidates by evaluating the putative small molecules' binding mode and providing a way to perform a ranking of vast compound libraries. There are however many peculiarities of ribonucleic acids that affect both performance and accuracy of docking protocols, and this makes its application challenging. The polyanionic backbone of RNA determines a peculiar charge distribution on the polymer surface—quite different from the one characterizing proteins—to which the scoring functions were traditionally calibrated (Disney, 2019). Furthermore, docking protocols do not explicitly consider the role of solvent during the molecular recognition process, whereas structural data have highlighted how water molecules can stabilize RNA-ligand complexes, often mediating hydrogen bonds networks (Fulle and Gohlke, 2009). However, the aspect that mostly affects RNA-docking accuracy is the flexibility and the dynamic behavior characterizing ribonucleic acids, which are usually neglected by docking algorithms, thus limiting the discovery of compounds targeting a narrow region of the conformational space (Hermann, 2002; Fulle and Gohlke, 2009; Disney et al., 2014). An attempt to overcome these limitations was conducted by Stelzer et al., who performed a docking-based virtual screening on an RNA dynamic ensemble constructed by combining molecular dynamics simulations (MD) with NMR spectroscopy and reported the discovery of six molecules able to bind HIV-1 TAR with quite good affinity. MD simulations would represent a valuable computational tool with which to investigate different ligand–RNA recognition processes, fully considering both target flexibility and the solvent presence. Interestingly, molecular mechanics force fields (FF), such as AMBER or CHARMM, were revisited and refined during the last year to improve ribonucleotide simulation accuracy (Pérez et al., 2007; Denning et al., 2011). Nevertheless, the use of MD is mostly limited to the fluctuation exploration in the post-docking procedure since ligand–target associations are rare events that can be sampled only through long-timescale computationally expensive simulations. An implementation of classical MD, called supervised molecular dynamics (SuMD), was recently developed in our research group. SuMD is able to speed up the exploration of the ligand–receptor recognition pathways on a nanosecond timescale through the implementation of a tabu-like supervision algorithm (Sabbadin and Moro, 2014). The protocol was so far validated in different scenarios, including ion–protein, ligand–protein, and peptide–protein bound complexes, proving that it could reproduce the experimental determined final state with great geometric accuracy (Cuzzolin et al., 2016; Salmaso et al., 2017; Bissaro et al., 2019).

In this work, SuMD simulations were applied for the first time to investigate the recognition mechanism involving ribonucleic acid macromolecules with the aim to extend the methodology applicability domain. This pilot study, which provided encouraging results, took into account a plethora of different ribonucleic complexes of pharmaceutical interest, the three-dimensional structures of which are known and available on the Protein Data Bank archive (Berman et al., 2000). SuMD methodology proved its ability in describing, with a reduced computational effort, the whole process of ligand–RNA recognition (from the unbound to the bound state), independently by the target topological complexity. As far as we know, this represents the first attempt to overcome methodological limitations within molecular docking when applied to ribonucleic acids, describing binding events through an all-atoms MD-based approach. This study confirms the possible use of SuMD as an innovative computational tool that can accelerate the discovery of new drug candidates and with peculiar attention to SMIRNAs.

## Materials and Methods

### Software Overview

MOE suite (Molecular Operating Environment, version 2018.0101) was used to perform most of the general molecular modeling operations, such as RNA and ligand preparation. All these operations have been performed on an 8 CPU (Intel® Xeon® CPU E5-1620 3.50 GHz) Linux workstation. Molecular dynamics (MD) simulations were performed with an ACEMD engine (Harvey et al., 2009) on a GPU cluster composed of 18 NVIDIA drivers whose models go from GTX 980 to Titan V. For all the simulations, the ff14SB force field with χ modification tuned for RNA (χOL3) was adopted to describe ribonucleic acids, while a general Amber force field (GAFF) was adopted to parameterize small organic molecules (Wang et al., 2006; Sprenger et al., 2015; Tan et al., 2018).

### Structures Preparation

The three-dimensional coordinates of each RNA–SMIRNA complex investigated were retrieved from the RCSB PDB database and prepared for SuMD simulations as herein described (Cuzzolin et al., 2016). For structures solved by NMR, which contain multiple conformations of the same complex, the one with the lowest potential energy (usually the first) was selected and then used. All complexes were then processed by means of an MOE protein structure preparation tool: missing atoms in nucleotide bases were built according to AMBER14 force field topology. Missing hydrogen atoms were added to X-Ray-derived complexes, and appropriate ionization states were assigned by Protonate-3D tool (Labute, 2009). Ligand coordinates (both small molecules and peptides) were moved at least 30 Å away from RNA binding cleft, a distance bigger than the electrostatic cut-off term used in the simulation (9 Å with Amber force field) to avoid premature interaction during the initial phases of the SuMD simulations.

### Solvated System Setup and Equilibration

Each system investigated by means of SuMD contained an RNA target macromolecule, and the respective ligand, which was a SMIRNA or a peptide, moved far away from the binding site as previously described. The systems were explicitly solvated by a cubic water box with cell borders placed at least 15 Å away from any RNA/ligand atom, using TIP3P as a water model. To neutralize the total charge of each system, Na^+^/Cl^−^ counterions were added to a final salt concentration of 0.154 M. The systems were energy minimized by 500 steps with the conjugate-gradient method, then 500,000 steps (1 ns) of NVT followed by 500,000 steps (1 ns) of NPT simulations were carried out, both using 2 fs as time step and applying harmonic positional constraints on RNA and ligand heavy atoms by a force constant of 1 kcal mol^−1^ Å^−2^, gradually reducing with a scaling factor of 0.1. During this step, the temperature was maintained at 310 K by a Langevin thermostat with low dumping of 1 ps^−1^ and the pressure at 1 atm by a Berendsen barostat (Berendsen et al., 1984; Loncharich et al., 1992). The M-SHAKE algorithm was applied to constrain the bond lengths involving hydrogen atoms. The particle-mesh Ewald (PME) method was exploited to calculate electrostatic interactions with a cubic spline interpolation and 1 Å grid spacing, and a 9.0 Å cutoff was applied for Lennard–Jones interactions (Essmann et al., 1995).

### Supervised Molecular Dynamics (SuMD) Simulations

**Figure 1:**
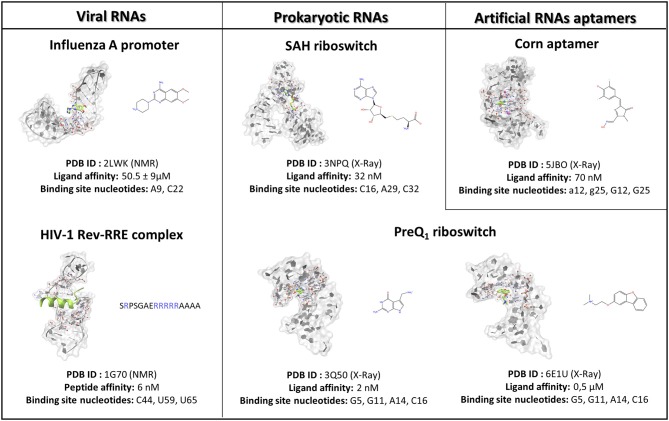
The case studies selected for the SuMD methodological validation are herein summarized and subdivided into RNA of viral origin, prokaryotic origin, or artificially engineered aptamers. For each complex investigated, the three-dimensional structure is depicted, representing with a green color the reference ligand, together with the nucleobases selected to define the binding site position in the SuMD simulations. Finally, the chemical structures of each ligand are reported, along with the experimental datum of binding affinity. In the case of the peptide, the primary sequence is reported, highlighting the basic residues constituting the arginine reach motif (ARM) in a blue color.

Molecular dynamics simulations represent a well-validated computational tool that, through the numerical solution of the Newton equation of motion, makes it possible to describe the time-dependent evolution of a molecular system. Despite the impressive temporal resolution characterizing the technique, to capture pharmaceutically relevant events, such as the molecular recognition between a drug and its biological target, huge computational efforts are required. The SuMD protocol instead improves the efficiency with which a binding event is sampled, from a microsecond to a nanosecond timescale, by applying a tabu-like algorithm. In detail, short (600 ps long) unbiased MD trajectories are collected, and these monitor, during the entire simulation, the distance between the ligand center of mass with respect to the ribonucleic acid binding site; then, those distance points are fitted into a linear function. Only productive MD steps in which the computed slope is negative are maintained, thus indicating a ligand approach toward the RNA binding site. Otherwise, the simulation is restarted by randomly assigning the atomic velocities from the previous set of coordinates. The supervision algorithm controlled the sampling until the distance between the ligand and the ribonucleic binding site dropped below 5 Å, at which point it was disabled, and a short classical MD simulation was performed, allowing the system to relax. For each case study, up to a maximum of 10 SuMD binding simulations were collected, of which only the best was thoroughly analyzed and discussed in the manuscript. A detailed report on SuMD protocol performance can be found in the Supplementary Material. The three-dimensional RNA structures investigated in this study, along with the nucleotides selected for the computation of the respective binding cleft center of mass, are reported in Figure 1. In this implementation, the SuMD code is written in Python programming languages and exploits the ProDy python package to perform the geometrical ligand–target supervision process (Bakan et al., 2011).

### SuMD Trajectory Analysis

All the SuMD trajectories collected were analyzed by an in-house tool written in tcl and python languages, as described in the original publication (Salmaso et al., 2017). Briefly, the dimension of each trajectory was reduced, saving MD frames at a 20 ps interval; each trajectory was then superposed and aligned on the RNA phosphate atoms of the first frames and wrapped into an image of the system simulated under periodic boundary conditions. The geometric performance of SuMD methodology was evaluated, and it computed the ligand RMSD (Root mean square deviation) along with the entire simulation with respect to the experimental resolved three-dimensional complex. Furthermore, the RMSD of RNA structures were computed on the P atoms of the backbone and plotted over time, and these can be viewed in the Supplementary Figures S1–S6A. A ligand–RNA interaction energy estimation during the recognition process was calculated using an MMGBSA protocol, as implemented in AMBER 2014, and it plotted MMGBSA values over time (Miller et al., 2012). The MMGBSA values were also arranged according to the distances between ligand and ribonucleic target mass centers in the Interaction Energy Landscape plots (Supplementary Figures S1–S6B). Here, the distances between mass centers are reported on the x-axis, while the MMGBSA values are plotted on the y-axis, and these are rendered by a colorimetric scale going from blue to red for negative to positive energetic values. These graphs allow for the evaluation of the variation of the interaction energy profile at different ligand–RNA distances; this helps to individuate meta-stable binding states during the binding process. Furthermore, for each target investigated in this work, the nucleotides within a distance of 4 Å from the respective ligand atoms were dynamically selected to qualitatively and quantitatively evaluate the number of contacts during the entire binding process. The most contacted nucleotides were thus selected, to compute a per-nucleotide electrostatic and vdW interaction, and energy contribution, with the ribonucleic target. NAMD was used for post-processing computation of electrostatic interactions using an AMBER ff14SB force field. The cumulative electrostatic interactions were computed for the same target nucleotides by summing the energy values frame by frame along the trajectory, and the resulting graphs were reported to the lower-right of movies provided as Supplementary Videos 1–6. Representations of the molecular structures were prepared with VMD software (Humphrey et al., 1996).

## Results and Discussion

To investigate the SuMD applicability domain and accuracy in the context of ribonucleic acid molecular recognition, a retrospective validation approach was selected, and it stressed the computational methodology ability in geometrically reproducing experimental binding modes of SMIRNA or small folded peptides. The three-dimensional structures of six ligand–RNA complexes solved both through X-Ray and NMR spectroscopy were retrieved from the RCSB PDB database and prepared for subsequent SuMD simulations moving ligands far away from the ribonucleic binding clefts, as accurately described in materials and methods section. The RNA structures, reported in Figure 1, were selected to span a vast plethora of pharmaceutically interesting ribonucleic targets, which vary between being of viral and bacterial origin, up to artificially engineered aptamers. Furthermore, the selected structures provide an overview of different peculiar three-dimensional RNA motifs, from a small stem-loop to a riboswitch characterized by a complex architecture. The results collected through SuMD simulations are then reported herein along with the geometric and interactives analysis performed. A summary of all the statistics regarding the simulation performances are reported in the Supplementary Information.

### Targeting Viral RNAs (vRNAs)

The discovery and design of new antiviral compounds targeting viral proteins are complicated by the enormous variability affecting these macromolecules, an aspect representing the core of the drug resistance phenomenon. On the other hand, lncRNA regions belonging to viral genomes, being less affected by genetic mutations and having no counterpart in human organisms, are becoming attractive pharmaceutical targets. Aminoglycosides, antibacterial drugs known to inhibit protein synthesis acting at the level of the prokaryotic ribosome, have proven to be promiscuous molecules that are also able to bind lncRNA structural elements of viral genomes (Bernacchi et al., 2007). This experimental evidence has paved the way for the discovery of drug-like small molecules able to inhibit the replication for a plethora of pathological viral diseases, such as human immunodeficiency virus (HIV), hepatitis C virus (HCV), severe respiratory syndrome coronavirus (SARS CoV), and influenza A virus (Hermann, 2016).

#### Influenza a Virus Promoter

Influenza A represents a group of viruses differing from virulence and pathogenicity profiles that all belong to the Orthomyxoviridae family. The Influenza A genome comprises eight negative-sense single-stranded RNA segments (vRNA) encoding for 13 proteins (Coloma et al., 2009). The 5′-end and 3′-end terminal portions of each vRNA segment in the physiological condition fold together in a partial duplex, forming an arrangement called a promoter, which controls RNA-dependent RNA polymerase (RdRp) recognition and, thus, genome transcription and replication (Desselberger et al., 1980). Since the promoter sequences are highly conserved among Influenza A viruses and marginally affected by genetic variation that can enhance the onset of drug resistance, they represent a promising pharmaceutical target. The Varani research group, exploiting an NMR-based fragments screening approach, has identified 6,7-dimethoxy-2-(1-piperazinyl)-4-quinazolinamine (DPQ) as a promising scaffold for antiviral drug development as it is able to bind the Influenza A promoter region with a low micromolar affinity (K_d_ 50.5 ± 9 μM) and is also able to inhibit the virus replication in a comparable range of concentration (Lee et al., 2014). The SMIRNA binding mode was experimentally elucidated by means of NMR, as depicted in Figure 1, confirming DPQ recognition within the RNA major groove at the (A-A)-U internal loop level.

**Figure 2:**
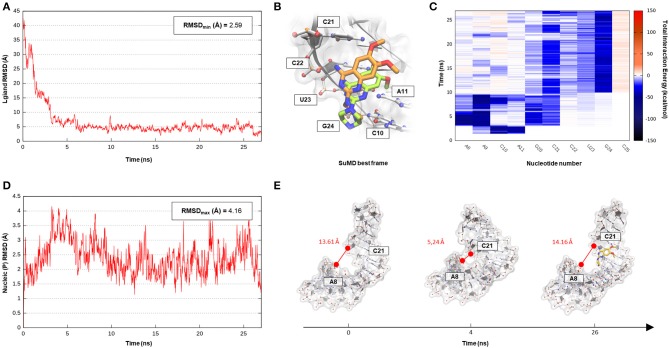
This panel summarizes the recognition pathway of the DPQ fragment with the Influenza A promoter region. **(A)** RMSD of DPQ heavy atoms against the PDB reference. **(B)** Superimposition between the experimental NMR complex (PDB ID 2LWK, green-colored DPQ molecule) and the SuMD conformation with lowest RMSD along the trajectory (orange-colored molecule). The nucleotides surrounding the binding site are reported. **(C)** Dynamic total interaction energy (electrostatic + vdW) computed for most contacted RNA nucleobase. **(D)** RMSD of RNA phosphate atoms belonging to the backbone, computed against the PDB reference. **(E)** Flexibility characterizing the RNA structure during DPQ binding event, binding clef dimension was monitored as the distance dynamically occurring between two key nucleotides (A8 and C21).

The SuMD algorithm was then applied to this first case study, in an attempt to investigate the entire DPQ binding mechanism, stressing at the same time the methodology accuracy in reproducing the experimental solved complex. A first interesting aspect is represented by the reduced time window of 30 ns required to sample a putative molecular recognition event between DPQ and its ribonucleic target (Supplementary Video 1). This result is quite impressive, especially if compared with classical MD simulations, which otherwise would require extensive computational efforts. At the end of the simulation, as depicted in the Figure 2 graph, the SMIRNA has converged both from a geometrical and interactive point of view toward the NMR structure binding mode. The low RMSD_min_ value of 2.6 Å, computed on DPQ heavy atoms, confirm, also in the case of nucleic acids, SuMD ability in predicting a reasonable binding hypothesis. This value must not be evaluated with excessive severity, having been calculated only with respect to one of the 16 conformations of the complex deposited on the PDB database. The solution NMR structure has indeed highlighted an important variability in the DPQ positioning within the RNA binding site, with an RMSD_max_, computed on ligand-heavy atoms of 1.4 Å. Moreover, this approach makes it possible to peek at the entire molecular recognition process and to not focus merely on the final state. Figure 2C reports a time-dependent analysis performed on the nucleotides most frequently contacted during the simulation, reporting their cumulative contribution to binding, which is defined as the sum of each nucleotide electrostatic and van der Waals (vdW) interaction energy. It is encouraging to note how the nucleotides that computationally have shown a primary role in stabilizing the DPQ complex (A9–A11 and C21–G24) also correspond to those that have experimentally experienced the greatest chemical shift perturbations during NMR experiments. In addition, as reported in Figures 2B,C and on Supplementary Figure S1, SuMD simulation allows us to decipher the different role played by aforementioned nucleotides, some of them (A9–A11) participating only during the early phases of SMIRNA recognition (until 10 ns) and the other (C21–G24) stabilizing the complex within the ribonucleic cleft (after 10 ns). These results appear even more interesting if we consider the high flexibility characterizing the small RNA duplex. Despite the reduced time window explored by SuMD methodology, the structure has indeed shown a relevant RMSD_max_ of 4.2 Å from the initial experimental coordinates (Figure 2D). In detail, after a few ns of simulation, the promoter duplex in the ligand-free form folds back on itself, and only DPQ binding allows the structure to return to the experimental linear conformation (Figure 2E). The same behavior was coherently captured also by NMR experiments, which previously highlighted how the RNA helical axis curvature changes upon ligand binding, enlarging the dimension of the binding cleft (Lee et al., 2014).

#### HIV-1 Rev-RRE Complex

**Figure 3:**
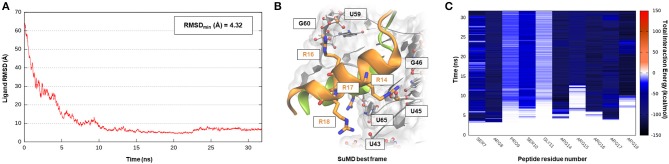
This panel summarizes the recognition pathway of RSG-1.2 peptide with HIV-1 REE. **(A)** RMSD of RSG-1.2 Cα atoms against the PDB reference. **(B)** Superimposition between the experimental NMR complex (PDB ID 1G70, green-colored peptide) and the SuMD conformation with lowest RMSD along the trajectory (orange-colored peptide). The nucleotides surrounding the binding site, along with R residues belonging to ARM, are reported. **(C)** Dynamic total interaction energy (electrostatic + vdW) computed for most contacted RNA nucleobase.

The human immunodeficiency virus of type 1 (HIV-1) is a retrovirus belonging to the Lentivirus family, and it is responsible for acquired immunodeficiency syndrome (AIDS). RNA–protein interactions play a fundamental role in controlling the HIV replication cycle and, consequently, virulence profile (Battiste et al., 1996). HIV-1 Rev, in particular, is a small regulatory protein that drives the nuclear export of unspliced and partially spliced viral mRNAs transcripts. Rev protein mediated its function, recognizing a purine-rich bulge within stem-loop IIb of the Rev response element (RRE), a highly structured mRNA region within an *env* intron (DiMattia et al., 2010). The minimal binding domain in the Rev protein is constituted by a short α-helix folded peptide, which contains an arginine-rich binding motif (ARM), a domain known to be important also for tat-TAR (trans-acting region) interactions in HIV. Harada et al., exploiting an *in-vivo* strategy, have identified a class of specific RNA-binding peptides able to target HIV-1 Rev-RRE complex. Specifically, RSG-1.2, an α-helical peptide of 22 amino acids, was selected among a combinatorial library and subsequently engineered, providing a 7-fold increase in binding affinity and a 15-fold increase in selectivity toward the ribonucleic target, further resulting in an *in vivo* ability to completely disrupt the RNA–Rev protein interaction (Harada et al., 1996, 1997). The solution structure of an oligonucleotide portion derived from HIV-1 RRE-IIb stem domain in a complex with an RSG-1.2 peptide was solved through NMR, providing structural details about vRNA targeting by means of the small peptide (Gosser et al., 2001). We have therefore chosen this case study to validate SuMD performance in one of the most complex methodological scenarios, namely the molecular recognition between two highly flexible partners: a small α-helix folded peptide and a portion of ribonucleic acid. In addition, the predominant electrostatic component that both characterizes the RNA polyanionic backbone and the small polycationic peptide, which contain six Arg residues, makes the prediction of the binding mode even more complex. Despite the unfavorable premises, a few tens of ns proved to be sufficient for the SuMD protocol to sample a binding hypothesis for the RSG-1.2 peptide. During the simulation, as observable on Supplementary Video 1, the peptide was accommodated with the correct orientation within the HIV-1 RRE-IIb major groove reaching, as reported in Figure 3A, an RMSD_min_ value of 4.3 Å, computed on Cα peptide atoms. Although the geometric accuracy is lower than the previous example, the SuMD simulation has allowed us to identify the main interactive hotspots stabilizing the complex. As hypothesized and confirmed by Figure 3C, the ARM motif plays a fundamental role in anchoring the RSG-1.2 peptide, with charged residue R 16, R17, and R18 mediating fork electrostatic interactions with the phosphate atoms of the ribonucleic backbone, in a coherent way with the experimentally solved structure. Furthermore, the analysis performed on the trajectory (Supplementary Figure S2) has highlighted the peculiar behavior of R14; its guanidinium side chain is deeply buried within the RNA groove, where, differently from the other charged residues, it stabilizes the peptide through a solvent-shielded hydrogen bond and vdW interactions, an aspect in great agreement with the experimental NMR data (Gosser et al., 2001).

### Targeting Prokaryotic RNAs

In the last decades, the discovery that many aminoglycoside compounds clinically exploited to treat severe bacterial infections mediated their action by affecting the ribosome machinery confirmed the initial hypothesis of considering RNA, especially prokaryotic ones, as an appetible pharmaceutical target (Disney, 2019). However, the drugs that target ribosomes represent an exception, rather than a model: the abundance of ribosome macromolecules in the cytoplasmic compartment means, therefore, that even modest drug-binding affinity could result in acceptable therapeutic efficacy (Warner et al., 2018). Apart from ribosomes, a putative regulatory role of lncRNAs in bacterial systems has recently become increasingly clear. From a mechanistic point of view, it is possible to distinguish regulatory RNAs acting in *trans*, either by base-pairing with a complementary region in the target mRNA or by sequestration of an RNA-binding protein and regulatory sequences that, in contrast, are encoded as part of the mRNA for the gene they regulate, thus acting in *cis* (Sherwood and Henkin). Riboswitches, which are structured elements typically found in the 5′ untranslated regions (UTR) of mRNAs, represent an interesting example of the latter case (Tucker and Breaker, 2005). These RNA elements, through an aptameric portion, directly sense a physiological signal (ions, cofactors, or metabolites) and transmit the information to the gene expression machinery via a signal-dependent RNA conformational change (Sherwood and Henkin, 2016). The discovery that clinically approved antibacterial Roseflavin exerts part of its therapeutic action by binding the flavin mononucleotide (FMN) riboswitch, together with the increasing availability of structural data on riboswitches, has made these targets very interesting pharmaceutically (Pedrolli et al., 2012).

#### S-Adenosylhomocysteine Riboswitch

**Figure 4:**
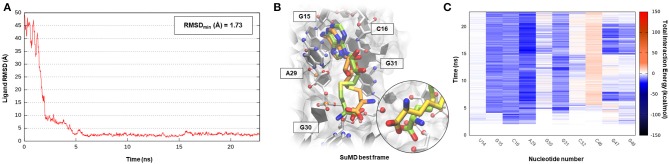
This panel summarizes the recognition pathway of the SAH molecule with SAH riboswitch. **(A)** RMSD of SAH heavy atoms against the PDB reference. **(B)** Superimposition between the experimental X-Ray complex (PDB ID 3NPQ, green-colored SAH molecule) and the SuMD conformation with lowest RMSD along the trajectory (orange-colored molecule). The nucleotides surrounding the binding site are reported. Within the circular window, the SuMD conformation sampled for SAH tail is compared to a different crystallographic reference (PDB ID 3NPN). **(C)** Dynamic total interaction energy (electrostatic + vdW) computed for most contacted RNA nucleobase.

S-adenosyl-(L)-methionine (SAM) is a fundamental cofactor that serves as the primary methyl group donor in a large set of biochemical reactions. In bacteria, SAM homeostasis is so important to the point that at least six classes of RNA riboswitch regulatory elements have since now been characterized (Weinberg et al., 2010). Following SAM-mediated methylation, the by-product S-adenosyl-(L)-homocysteine (SAH) that is released, due to its high toxicity, must be readily degraded by SAH hydrolase (ahcY) enzymes. Recently, a new type of riboswitch was discovered, and it is able to sense and be responsible for the intracellular SAH concentration, upregulating the expression of ahcY enzymes in prokaryotes (Wang et al., 2008). The aptameric portion of the SAH riboswitch recognizes its cognate ligand with a quite high binding affinity of 32 nM and, surprisingly, also provides a discrete selectivity profile toward the original cofactor SAM (1,000-fold lower affinity), ensuring a fine regulation of the SAM/SAH metabolic cycle. The high-resolution crystal structure of the SAH riboswitch aptameric domain in complex with its cognate ligand was recently solved, elucidating the molecular basis for SAH substrate specificity (Edwards et al., 2010). This case study not only represents a pharmaceutical appealing prokaryotic RNA target but also provides the opportunity to stress the SuMD performance in a more complex binding site recognition, if compared to the simple duplex structures until now investigated. The SAH molecule indeed binds a small cleft located in the minor groove of the SAH riboswitch, which adopts an unusual “LL-type” pseudoknot conformation. Also, in this case, around 20 ns were sufficient for the SuMD protocol to sample a putative molecular recognition trajectory (Supplementary Video 3). In detail, as reported in Figure 4, after only a few nanoseconds, SAH reached the riboswitch binding cleft reproducing the crystallographic complex with a notable geometric accuracy (RMSD_min_ 1.7 Å). Then, the ligand conformation remained stable until the end of the simulation. From an interactive point of view, as reported in Figure 4C and also in Supplementary Figure S3, the SuMD trajectory analysis correctly highlighted the stabilizing role played by nucleotide C16 and A29, among which the adenine core of SAH is intercalated, providing the greatest vdW interactions. In contrast, the electrostatic contribution to binding analysis has revealed a divergent situation. Indeed, nucleobase G15, mediating a hydrogen bond network with an SAH adenine scaffold, is responsible for a great stabilizing contribution, whereas nucleotide C46 has shown during the entire simulation an unexpected repulsive contribution. The reason for this can be found in the conformation sampled by SuMD for the SAH homocysteine terminal tail. As depicted by Figure 4B, the carboxylic moiety of the ligand spatially approaches the C46 pyrimidine carbonyl, whereas in the crystallographic structure (green representation), through a simple bond rotation, the interaction is instead mediated by the vicinal amino group. Curiously, the same research group also deposited on the PDB database a worst resolution structure of the complex under investigation (PDB ID 3NPN), reporting the same apparently energetic unfavored SAH conformation described by the SuMD protocol (Figure 4B, circular window), thus validating the goodness of the sampling and the flexibility characterizing the ligand tail.

#### Pre-queuosine_1_ Riboswitch

**Figure 5:**
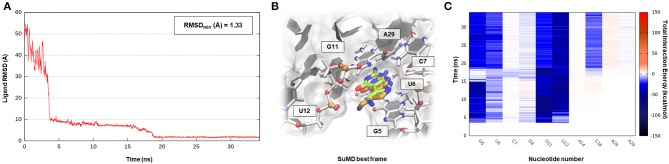
This panel summarizes the recognition pathway of the PreQ_1_ molecule with PreQ_1_-1 riboswitch. **(A)** RMSD of PreQ_1_ heavy atoms against the PDB reference. **(B)** Superimposition between the experimental X-Ray complex (PDB ID 3Q50, green-colored PreQ_1_ molecule) and the SuMD conformation with lowest RMSD along the trajectory (orange-colored molecule). The nucleotides surrounding the binding site are reported. **(C)** Dynamic total interaction energy (electrostatic + vdW) computed for most contacted RNA nucleobase.

Pre-queosine_1_ (PreQ_1_), or 7-aminomethyl-7-deazaguanine, is a metabolic intermediate in the synthetic pathway that, starting from guanosine-5′-triphosphate (GTP) nucleotide, originates the hypermodified guanine derivate queuosine (Q). Q has been detected both in eubacteria and eukaryotic organisms where it occupies the anticodon wobble position of tRNAs specific for the amino acid asparagine, aspartate, histidine, and tyrosine (Roth et al., 2007). Q modification has been related to an improvement in translation fidelity as well as bacterial pathogenicity. Interestingly, only prokaryotes can synthesize Q via a multistep reaction, whereas eukaryotes are obliged to assimilate the nucleoside through the diet (Eichhorn et al., 2014). In bacteria like *Bacillus subtilis (Bs)* or *Thermoanaerobacter tengcongenesis (Tt)*, the expression of genes responsible for Q biosynthesis is negatively modulated by the intermediate PreQ_1_ intracellular concentration. PreQ_1_, binding to a small aptameric RNA motif composed of 34 nucleotides determines the folding of the PreQ_1_ riboswitch in an “H-type” pseudoknot structure in which more than half of the nucleobases engage in triplet or quartet interactions (Rieder et al., 2010; Jenkins et al., 2011). The three-dimensional structure of the class I PreQ_1_ riboswitch in complex with its cognate ligand was solved by X-ray crystallography (PDB ID 3Q50), and this allowed us to speculate about the quite impressive binding affinity characterizing this endogenous precursor (K_d_ = 2 nM) (Edwards et al., 2010). Even in this case, <40 ns of SuMD simulation proved to be sufficient in describing a binding event between the metabolic intermediate PreQ_1_ and its related riboswitch (Supplementary Video 4). As observable in Figure 5A, PreQ_1_ recognition mainly articulates in three well-distinguishable phases. In the beginning, the ligand approaches the riboswitch binding site vestibule where it negotiates for about 15 ns the accommodation in the deep cleft before converging, with great geometric accuracy (RMSD_min_ 1.3 Å), toward the solved crystallographic conformation. This behavior has also been captured by the interaction energy graph (Supplementary Figure S4B), highlighting the presence of two major sites visited during the recognition trajectory, i.e., the canonical binding cleft and the aforementioned external vestibular region, located about 10 Å apart. It is interesting to note the comparable interaction energy characterizing these two distal sites, which are distinguishable for their different degrees of solvent exposition. In addition, the dynamic interaction fingerprint reported in Figure 5C, elucidates the role played by the binding site nucleotides during recognition in a coherent way with respect to the results reported on the original publication.

**Figure 6:**
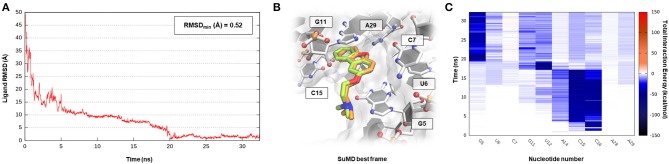
This panel summarizes the recognition pathway of HMJ molecule with PreQ_1_-1 riboswitch. **(A)** RMSD of HMJ heavy atoms against the PDB reference. **(B)** Superimposition between the experimental X-Ray complex (PDB ID 6E1U, green-colored HMJ molecule) and the SuMD conformation with lowest RMSD along the trajectory (orange-colored molecule). The nucleotides surrounding the binding site are reported. **(C)** Dynamic total interaction energy (electrostatic + vdW) computed for most contacted RNA nucleobase.

All the cases considered so far have confirmed the ability of SuMD to predict reasonable binding hypotheses for different ligands when exploiting as starting point the experimental structures of the ribonucleic targets in which each of these ligands were originally co-crystallized. From a pharmaceutical and applicative perspective, however, it is often required to rationalize the binding mode of compounds that are in most of the cases different from the ones now co-crystallized. It has thus become crucial to understand how the choice of the initial RNA target conformation could affect SuMD performance. The studies performed by the Schneekloth Jr. group in the attempt to experimentally asses the druggability profile of PreQ_1_-I riboswitch through synthetic organic molecules have then given us an opportunity to further explore this question. In a recent scientific work, it the discovery of HMJ was indeed reported; this is a dibenzofuran derivative that, despite the not obvious chemical similarity with PreQ_1_, exhibits a sub-micromolar affinity to the RNA target (K_d_ = 0.5 μM) and the ability to induce premature transcriptional termination (Connelly et al., 2019). The three-dimensional structure determination of the complex was, however, quite difficult and was achieved only by designing a hybrid riboswitch aptamer sequence in which the nucleobase A14, as well as the two vicinal ones, were removed (PDB ID 6E1U). Since this structure lacked a key binding site nucleotides, it represent a non-optimal starting point for a computational study; we therefore decided to investigate the HMJ binding mechanism, exploiting the high-quality riboswitch structure originally solved in the presence of PreQ_1_ and then comparing the accuracy of the prediction with the experimental solved data. Encouragingly, even for such a system, the SuMD protocol has succeeded in sampling, in about 30 ns, an extremely accurate binding hypothesis for HMJ, whose RMSD_min_ was computed with respect to reference structure (PDB ID 3Q50) and has reached the impressive value of 0.5 Å (Figure 6A, Supplementary Video 5). From the analysis of the trajectory, it was furthermore possible to confirm how the benzofuran ligand competes with PreQ_1_ for the riboswitch binding site. As depicted by Figure 6C, and as is coherent with experimental evidence, HMJ makes a strong stabilizing interaction with the nucleobases G5, G11, and C16, which define the “floor” and the “ceiling” of the binding cleft where the aromatic core stacks, and nucleobase U6, C15, and A29, which shape instead the binding cavity borders. Moreover, the Interaction Energy Landscape (Supplementary Figure S5B) highlights a binding profile similar to the one previously described for the cognate ligand PreQ_1_, confirming the vestibular region's role in recruiting the riboswitch binding partners.

### Targeting Artificial RNA Aptamers Containing G-Quadruplex Motifs

The discovery, made in 1994, that the green fluorescent protein (GFP) from the jellyfish *Aequorea victoria* could be used as a marker for protein localization and expression has revolutionized molecular biology to the point that, in 2008, the discovery earned a Nobel prize (Swaminathan, 2009). However, since a minimal portion of the human genome is translated into proteins while most of it is transcribed into RNA, being able to investigate the dynamic and spatial properties of the human transcriptome has become essential. As there are no known naturally fluorescent RNAs, a series of *in vitro* engineered ribonucleic tags able to fold into peculiar three-dimensional structures were selected (Trachman and Ferré-D'Amaré, 2019). These RNAs, through an aptameric domain, can bind fluorophore molecules, increasing their spectroscopic signal and hence allowing for the dynamic monitoring of nucleic acid expression and localization in cells. Most of the fluorophore RNA binding sites, despite the different overall architecture, have evolutionarily converged on G-quadruplex motifs, supporting their important role in enhancing the fluorescence phenomenon, in a similar way to how the β-barrel domains characterize GFPs (Warner et al., 2014).

#### Corn Aptamer

**Figure 7:**
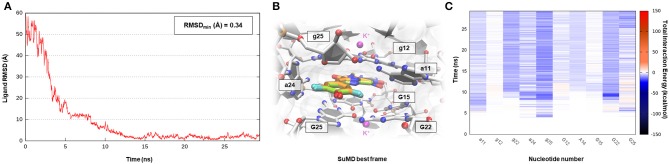
This panel summarizes the recognition pathway of DFHO molecules with the Corn aptamer. **(A)** RMSD of DFHO heavy atoms against the PDB reference. **(B)** Superimposition between the experimental X-Ray complex (PDB ID 5BJO, green-colored DFHO molecule) and the SuMD conformation with lowest RMSD along the trajectory (orange-colored molecule). The nucleotides surrounding the binding site are reported. **(C)** Dynamic total interaction energy (electrostatic + vdW) computed for most contacted RNA nucleobase.

Corn is one recently developed RNA aptamer engineered *in vitro* to bind 3,5-difluoro-4-hydroxybenzylidene imidazolinone-2-oxime (DFHO), a fluorophore analogous of red fluorescent protein (RFP) (Warner et al., 2017). Corn-DFHO differs from other similar RNA tags for its limited light-induced cytotoxicity, its minimal background fluorescence, and its increased photostability, thus representing a valuable imaging tool. Corn aptamer is characterized by an atypical three-dimensional structure elucidated by X-ray crystallography and biophysical experiments. How it is observable in Figure 1 that two RNA segments join together in a quasi-symmetric homodimer structure (1:2 chromophore:RNA stoichiometry) at the interfaces where a single DFHO molecule is tightly bound (K_d_ = 70 nM), stacking between two G-quadruplex planes stabilized by the presence of K^+^ ions (Warner et al., 2017). Despite the lack of therapeutic application for this aptamer, which is instead more suitable for molecular biology studies, the investigation of such a complex binding site recognition can be considered as a proof of concept to validate G-quadruplex motif targeting through an SuMD approach. Nucleotide quartet structures, which presence have been extensively characterized in the telomeric terminal portion of eukaryotes chromosomes and within gene promoter regions, are indeed acquiring increasing attention, as they could represent promising pharmaceutical targets (Balasubramanian and Neidle, 2009). As shown in Supplementary Video 6, SuMD methodology has produced a putative binding trajectory for DFHO in <30 ns, converging with an impressive geometrical accuracy toward the experimental solved complex (RMSD_min_ 0.34 Å) (Figure 7A). Moreover, the Dynamic Total Interaction Energy plot reported on Figure 7C, strongly retraces the interactive pattern already described on the original scientific work, highlighting the role played by nucleotide G12, G25 (first protomer), and g25 (second protomer) in circumscribing the sandwich cavity within which the aromatic chromophore stacks. Nucleobase A14 (first protomer) and a11 (second protomer) instead mediated a hydrogen bond network with oxime and imine moieties of the DFHO ligand, respectively. SuMD simulation has also illuminated how the entire binding process is not driven by the electrostatic contribution, as often it happens for SMIRNA, but is instead controlled by the vdW interactions (Supplementary Figure S6). From this perspective, Corn aptamer represents an unusual, but potentially revolutionary case study, as it distorts an old paradigm that has now since affected the identification of putative RNA binders. DFHO has indeed demonstrated how even apolar or anionic molecules can target ribonucleic acids reaching a nanomolar binding affinity. This provides the opportunity to expand the chemical space explorable by SMIRNA beside that of the well-known, but often problematic, polycationic compounds.

## Conclusion

Over the last decades, among all the biological macromolecules, proteins have represented the target of choice for the development of new drug candidates. Nucleic acids, on the other hand, have so far represented a less attractive target due to the difficulty in guaranteeing a selective recognition mechanism. The recent discovery of peculiar and physiologically stable three-dimensional conformation characterizing RNAs oligomers has, however, paved the way for the investigation of SMIRNA. The increasing availability of structural data for a wide range of relevant therapeutic ribonucleic targets has promoted the application of well-validated SBDD computational approaches, such as molecular docking, also in this field. However, the remarkable flexibility and the peculiar electrostatic potential, which distinguish nucleic acids from proteins, have readily highlighted the limitation of many of these methodologies. MD simulations would allow us to overcome some of the aforementioned problems; however, the computational cost required to capture rare events such as ligand binding has so far limited their routine utilization.

In this work, we have investigated the applicability domain of SuMD in the field of pharmaceutically relevant RNA polymers. The performances of the protocol were measured as the geometrical accuracy, expressed in terms of RMSD, with which an experimentally solved complex is predicted by the SuMD simulation. Case studies in this research were chosen in such a way as to span very different ribonucleic secondary, tertiary, and even quaternary structures, starting from small duplex stem-loops up to pseudoknot or aptameric homodimers, which contain G-quadruplex motifs. Furthermore, the recognition of different ligands was investigated, both small organic molecules and folded α-helical peptides.

Although this work must be considered as a preliminary investigation and the number of examples taken into consideration cannot guarantee statistical robustness, it is encouraging to note how, in all the six ribonucleic complexes simulated, SuMD correctly reproduced the experimentally solved final state starting from the unbound state in few hours of simulation. The accuracy of the protocol varies significantly in a system-dependent manner, but, in all the cases, it was possible to collect valuable interactive and energetic information about the nucleotides dynamically involved in the recognition process. Curiously, the RNA target in which the architecture of the binding site is not very complex, such as the stem-loop domain of Influenza A promoter and HIV-1 RRE, are those in which the computational protocol experienced the poorest geometric accuracy in reproducing the ligand-binding mode. A separate consideration must be made for the latter complex (PDB ID 1G70) since the recognition between two extremely flexible entities, i.e., the small peptide and the RNA duplex, represents a very challenging case. However, the results obtained, with an RMSD_min_ lower than 5 Å, are in line with those previously described when applying SuMD methodology to peptide–protein recognition. Moving toward more complex binding sites, such as the one that characterizes pseudoknot riboswitch structures or G-quadruple-shaped clefts, the geometric accuracy of the method progressively improves, with the best results obtained in the artificial aptameric structure (RMSD_min_ 0.34 Å). These findings are in agreement with a recent perspective work that assessed how the complexity of an RNA binding site, measured in terms of information content, could represent a valuable discriminant to individuate druggable oligonucleotides (Warner et al., 2018). Indeed, the three-dimensional complexity of a binding site makes ribonucleic pocket more similar to a protein-like environment rather than an ordered and repetitive structure like that characterizing DNA.

Furthermore, the high conformational flexibility that has characterized all the investigated ribonucleic structures (RMSD computed on RNA backbone are reported on Supplementary Material) during SuMD simulations has evidenced the importance of adopting techniques able to consider the flexibility of both macromolecules and ligands to better describe such complex molecular recognition. In conclusion, we have shown how SuMD can be a valid computational method to generate binding hypothesis for ribonucleic targets in a nanosecond timescale, explicitly considering both the role of the solvent and the flexibility of the macromolecule. SuMD simulation results could not only help with the interpretation and investigation of the complex mechanism of recognition characterizing SMIRNA, especially when structural information is not available, but they could also guide the rational discovery and optimization of these compounds.

## Data Availability Statement

The raw data supporting the conclusions of this article will be made available by the authors, without undue reservation, to any qualified researcher.

## Author Contributions

MB carried out the experiment. MB wrote the manuscript with support from MS. MS and SM supervised the project. MB and SM conceived the original idea.

### Conflict of Interest

The authors declare that the research was conducted in the absence of any commercial or financial relationships that could be construed as a potential conflict of interest.
