# Supplementary material for: Exploring the RNA-Recognition Mechanism Using Supervised Molecular Dynamics (SuMD) Simulations: Toward a Rational Design for Ribonucleic-Targeting Molecules?
Source: Front Chem. 2020 Feb 27;8:107. doi: 10.3389/fchem.2020.00107 (PMC7057144; doi:10.3389/fchem.2020.00107)
Supplement: Supplementary file 1 [file Data_Sheet_1.docx]

Supplementary Material

# Supplementary Data and Figures

SuMD replicas have been extensively analyzed through a proprietary tool, able to perform in a fully automated way a series of geometric and energetic analysis of the trajectories. Results for representative simulations are herein reported (grouped in panel).

| **Ribonucleic Systems** | **PDB ID** | **Figure number** |
| --- | --- | --- |
| Influenza A promoter | 2LWK | S1 |
| HIV-1 Rev-REE complex | 1G70 | S2 |
| SAH riboswitch | 3NPQ | S3 |
| PreQ_1_ riboswitch | 3Q50 | S4 |
| PreQ_1_ riboswitch | 6E1U | S5 |
| Corn aptamer | 5JBO | S6 |

In detail, for each representative replica six graphs are reported, summarizing:

1. The RNA RMSD computed on backbone P atoms. The RNA RMSD computed on backbone P atoms.
2. A ligand-RNA interaction energy estimation during the recognition process calculated using MMGBSA protocol as implemented in AMBER 2014, plotting MMGBSA values over time. The MMGBSA values are arranged according to the distances between ligand and ribonucleic target mass centers in the Interaction Energy Landscape plots. The distances between mass centers are reported on the x-axis, while the MMGBSA values on the y-axis, and are rendered by a colorimetric scale going from blue to red for negative to positive energetic values.
3. Per-residue decomposition of ligand-RNA electrostatic interaction energy (kcal/mol), with the aim to quantitatively characterize the role played by different nucleotides during molecular recognition.
4. Per-residue decomposition of ligand-RNA vdW interaction energy (kcal/mol), with the aim to quantitatively characterize the role played by different nucleotides during molecular recognition.


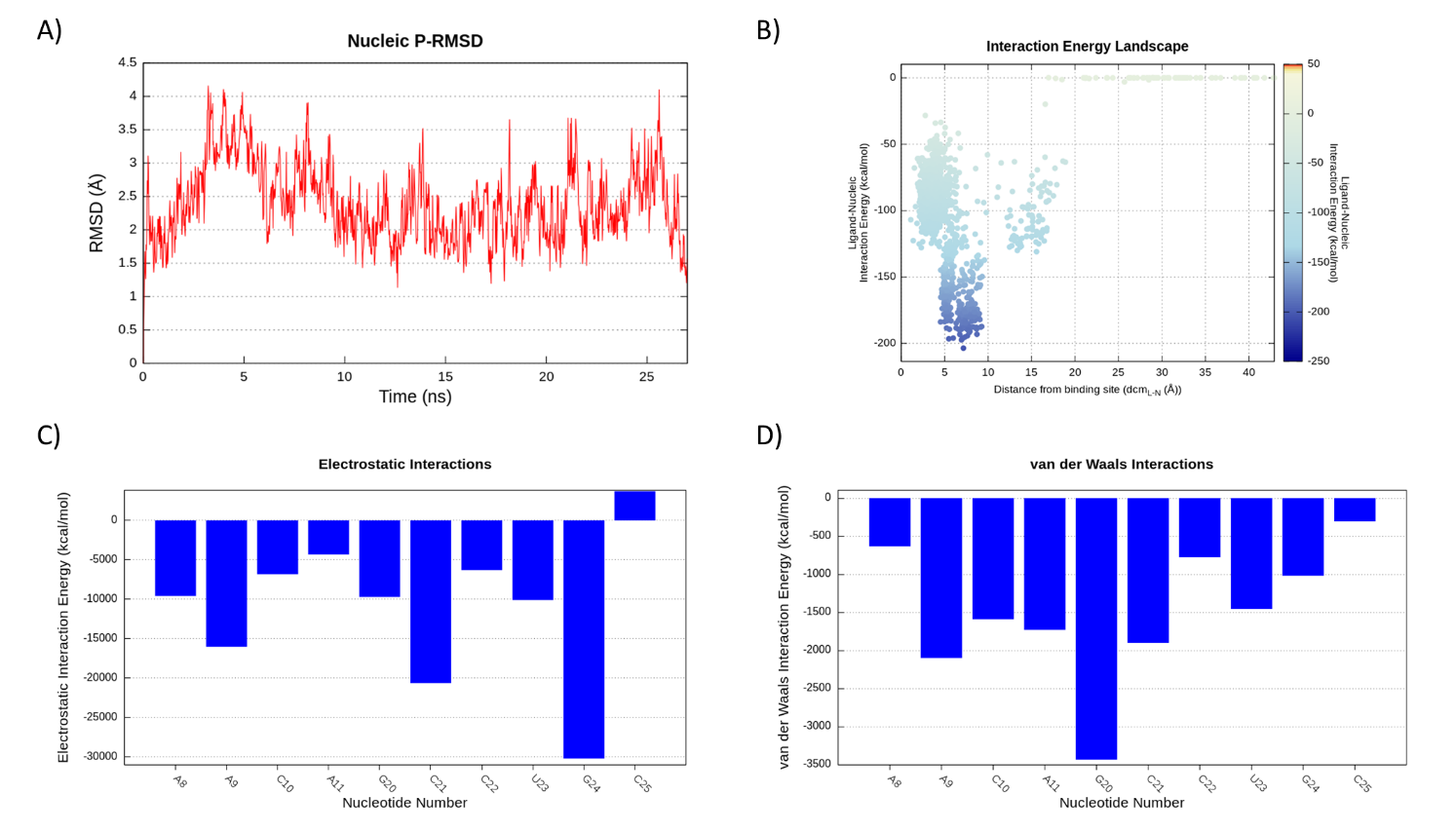


**Supplementary Figure S1:** SuMD simulation analysis on influenza A promoter (PDB ID 2LWK).


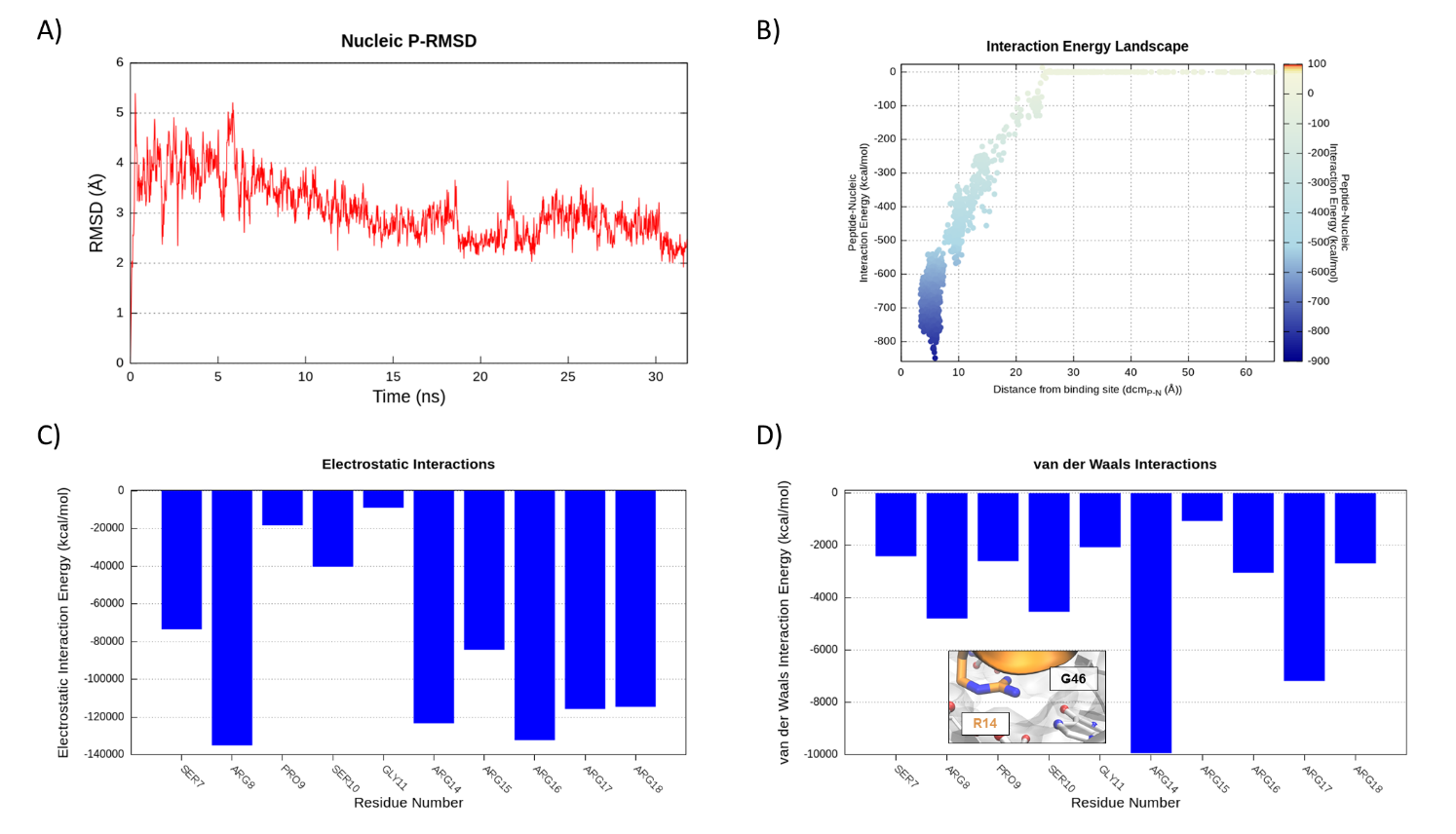


**Supplementary Figure S2:** SuMD simulation analysis on HIV-1 Rev-REE complex (PDB ID 1G70).


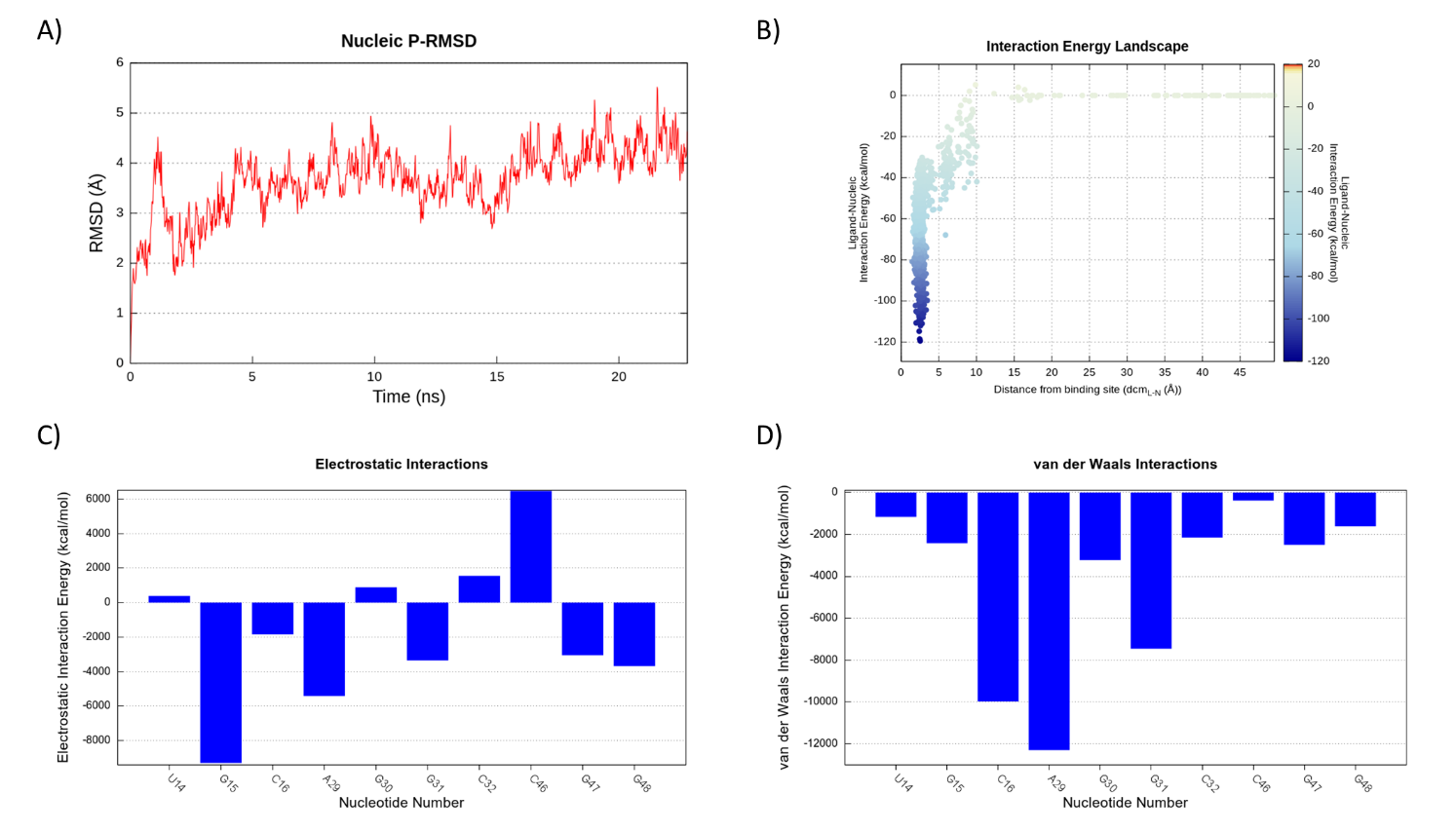


**Supplementary Figure S3:** SuMD simulation analysis on SAH riboswitch (PDB ID 3NPQ).


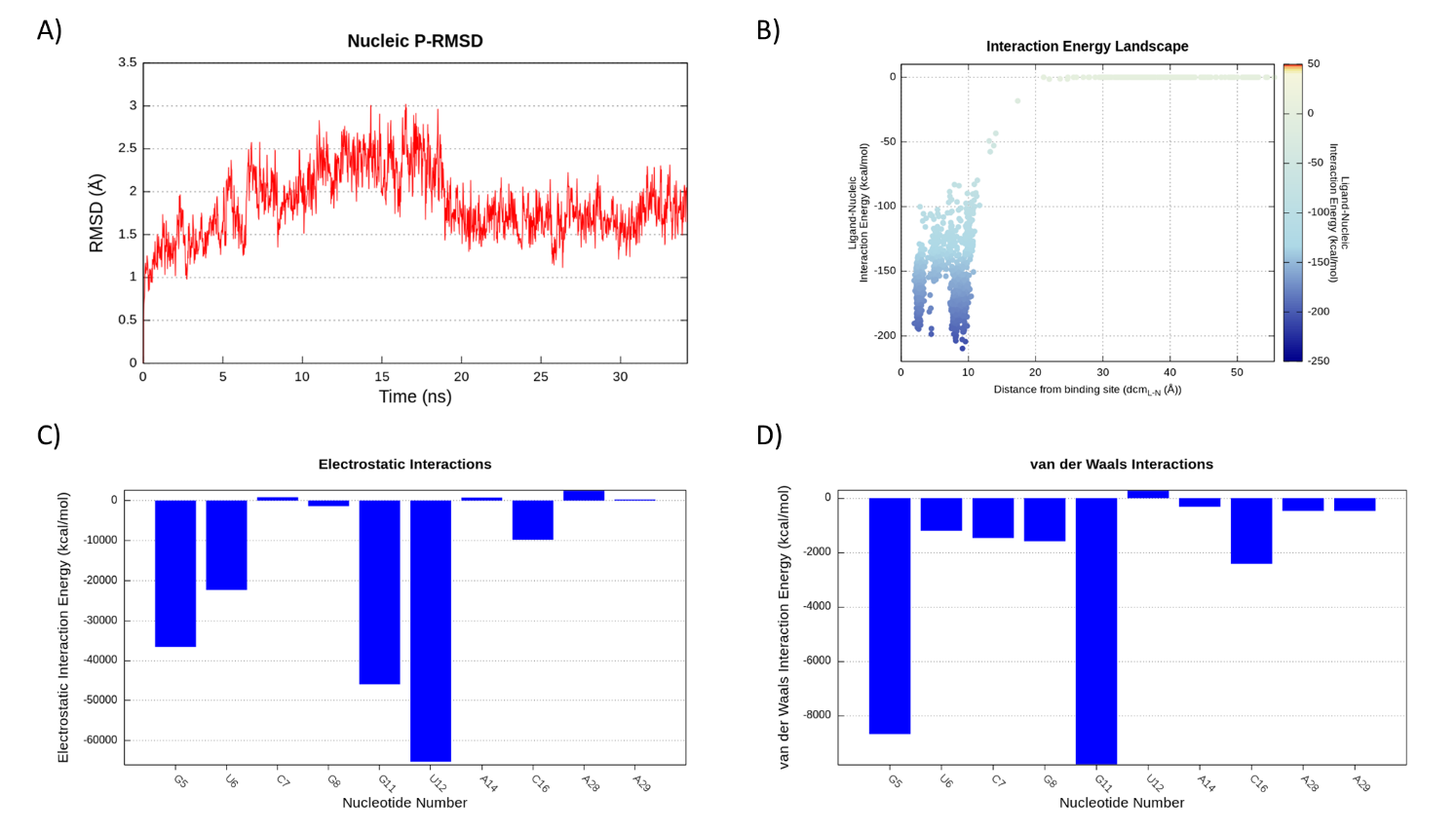


**Supplementary Figure S4:** SuMD simulation analysis on PreQ_1_ riboswitch (PDB ID 3Q50).


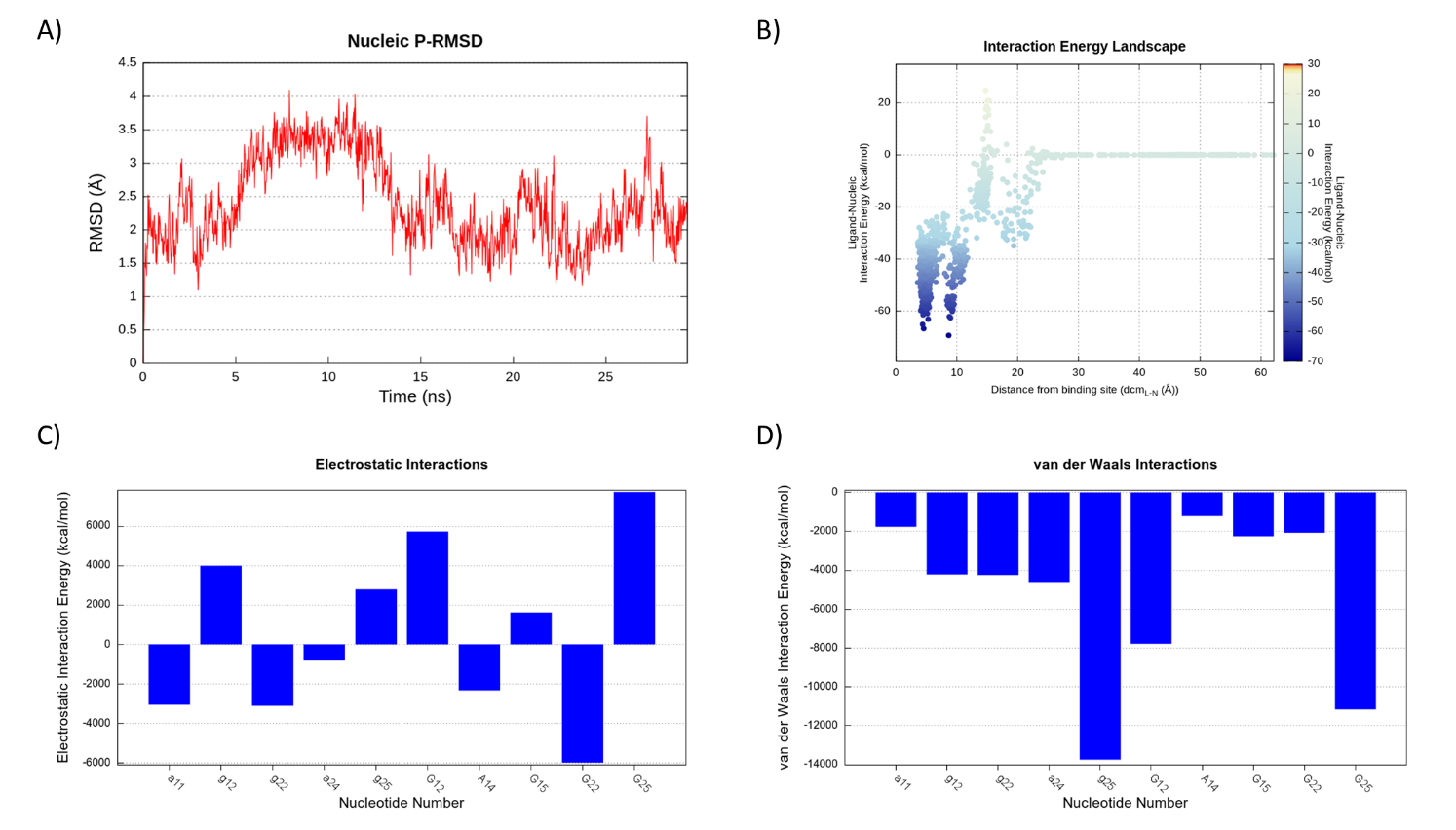


**Supplementary Figure S5:** SuMD simulation analysis on PreQ_1_ riboswitch (PDB ID 6E1U).


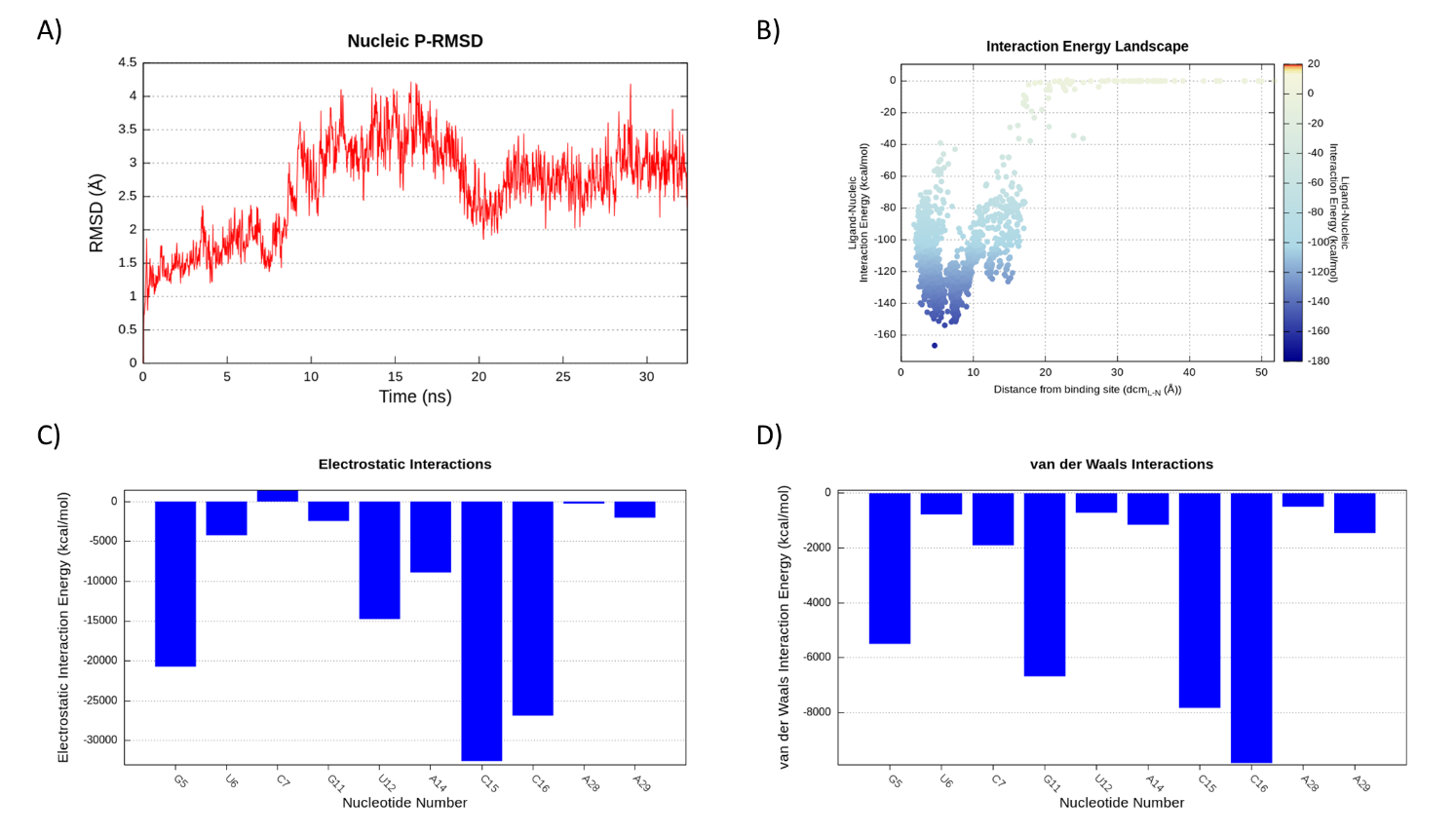


**Supplementary Figure S6:** SuMD simulation analysis on Corn Aptamer (PDB ID 5JBO).

# Supplementary Video

Each video is composed of four synchronized and animated panels that depict the molecular trajectory obtained by the SuMD simulation considering different aspects of the simulation. The time evolution is reported in a ns scale. In the first panel (upper-left), the molecular representation of the ribonucleic system is shown. The RNA backbone is represented by the ribbon style (silver colour) and the residues within 4 Å of each ligand investigated are shown, in white colour. Ligands references are rendered by green carbon atoms, while the ligands evaluated through SuMD simulation are rendered by orange carbon atoms. In the second panel (upper-right), the dynamic distance of each ligand center of mass (CM) from the respective RNA binding site during the trajectory is reported. In the third panel (lower-left), the MMGBSA energy profile is reported. The animated red circle highlights the value of the corresponding frame. The trend is depicted by a continuous black line obtained by smoothing the raw data (grey circles) using a Bezier curve procedure. In the fourth panel (lower-right) cumulative electrostatic interactions are reported for the 15 RNA residues most contacted by each ligand during the whole simulation.

| **Ribonucleic Systems** | **Video number** |
| --- | --- |
| Influenza A promoter | V1 |
| HIV-1 Rev-REE complex | V2 |
| SAH riboswitch | V3 |
| PreQ_1_ riboswitch | V4 |
| PreQ_1_ riboswitch | V5 |
| Corn aptamer | V6 |

# Supplementary on SuMD performances

For each case study examined, the following table shows the number of atoms composing the simulated system, the length of the simulation expressed both as SuMD time (total of productive MD steps) and as MD wall time (total of productive and rejected MD steps). Furthermore, SuMD performances computed, using as reference an NVIDIA GTX 1080Ti graphic card, have been herein reported.

| **Ribonucleic Systems** | **Simulated atoms** | **SuMD time  (ns)** | **MD wall time (ns)** | **Performance (ns/day)** |
| --- | --- | --- | --- | --- |
| Influenza A promoter | 66944 | 27 | 61 | 102 |
| HIV-1 Rev-REE complex | 80339 | 32 | 47 | 92 |
| SAH riboswitch | 70801 | 23 | 26 | 103 |
| PreQ_1_ riboswitch | 47286 | 34 | 70 | 141 |
| PreQ_1_ riboswitch | 48514 | 32 | 61 | 139 |
| Corn aptamer | 103591 | 29 | 59 | 67 |
